# Optical Coherence Tomography Angiography (OCTA) Captures Early Micro-Vascular Remodeling in Non-Melanoma Skin Cancer During Superficial Radiotherapy: A Proof-of-Concept Study

**DOI:** 10.3390/diagnostics15212698

**Published:** 2025-10-24

**Authors:** Gerd Heilemann, Giulia Rotunno, Lisa Krainz, Francesco Gili, Christoph Müller, Kristen M. Meiburger, Dietmar Georg, Joachim Widder, Wolfgang Drexler, Mengyang Liu, Cora Waldstein

**Affiliations:** 1 Department of Radiation Oncology, Comprehensive Cancer Center Vienna, Medical University Vienna, 1090 Vienna, Austria; 2 PolitoBIOMed Lab, Department of Electronics and Telecommunications, Politecnico di Torino, 10129 Torino, Italy; fra.gili@studenti.polito.it (F.G.);; 3 Center for Medical Physics and Biomedical Engineering, Medical University Vienna, 1090 Vienna, Austria; 4 Department of Dermatology, Medical University Vienna, 1090 Vienna, Austria

**Keywords:** optical coherence tomography angiography, vascularization, radiotherapy, radiomics, non-melanoma skin cancer

## Abstract

**Background/Objectives**: This proof-of-concept study evaluated whether optical coherence tomography angiography (OCTA) can non-invasively capture micro-vascular alterations in non-melanoma skin cancer (NMSC) lesions during and after superficial orthovoltage radiotherapy (RT) using radiomics and vascular features analysis. **Methods**: Eight patients (13 NMSC lesions) received 36–50 Gy in 6–20 fractions. High-resolution swept-source OCTA volumes (1.1 × 10 × 10 mm^3^) were acquired from each lesion at three time points: pre-RT, immediately post-RT, and three months post-RT. Additionally, healthy skin baseline was scanned. After artifact suppression and region-of-interest cropping, (i) first-order and texture radiomics and (ii) skeleton-based vascular features were extracted. Selected features after LASSO (least absolute shrinkage and selection operator) were explored with principal-component analysis. An XGBoost model was trained to classify time points with 100 bootstrap out-of-bag validations. Kruskal–Wallis tests with Benjamini–Hochberg correction assessed longitudinal changes in the 20 most influential features. **Results**: Sixty-one OCTA volumes were analyzable. LASSO retained 47 of 103 features. The first two principal components explained 63% of the variance, revealing a visible drift of lesions from pre- to three-month post-RT clusters. XGBoost achieved a macro-averaged AUC of 0.68 ± 0.07. Six features (3 texture, 2 first order, 1 vascular) changed significantly across time points (adjusted *p* < 0.05), indicating dose-dependent reductions in signal heterogeneity and micro-vascular complexity as early as treatment completion, which deepened by three months. **Conclusions**: OCTA-derived radiomic and vascular signatures tracked RT-induced micro-vascular remodeling in NMSC. The approach is entirely non-invasive, label-free, and feasible at the point of care. As an exploratory proof-of-concept, this study helps to refine scanning and analysis protocols and generates knowledge to support future integration of OCTA into adaptive skin-cancer radiotherapy workflows.

## 1. Introduction

Non-melanoma skin cancer (NMSC) is the most common malignancy in the Caucasian population, with rising incidence rates especially in aging demographics. Radiotherapy (RT) offers tumor control rates comparable to surgery, often providing a valuable alternative for patients unsuited to surgical intervention [1]. However, a key limitation of RT is the inability to perform a complete histological tumor assessment. Consequently, treatment efficacy is typically evaluated using dermatoscopy, which may lack the sensitivity needed to detect subtle morphological changes in early stages [2].

Optical coherence tomography (OCT) is a non-invasive, label-free imaging modality that generates high-resolution volumetric images of tissue in vivo [3]. Its utility in dermatology lies in the detailed visualization of skin layers and structures such as sweat ducts. A functional extension of OCT, known as OCT angiography (OCTA), can provide background-free vasculature maps without additional hardware modifications [4]. By capturing microvascular changes correlated with disease progression and treatment response, OCTA has emerged as a promising tool for dermatological applications—particularly in identifying and monitoring basal cell carcinoma, melanoma, and chronic venous insufficiency [5,6,7]. More recently, OCTA has shown utility in assessing how RT impacts the tumor microenvironment, including vascular, stromal, and immunological components that govern both treatment efficacy and resistance [8]. Early, dose-dependent vascular alterations have been observed in irradiated tissue, often preceding explicit clinical changes [9,10,11], opening the door for adaptive RT strategies. In addition, OCTA can differentiate keratinocyte carcinoma subtypes [12] and improve lesion segmentation through automated techniques [13], underscoring its potential role in enhancing diagnostic precision and treatment monitoring.

While OCT’s limited penetration depth generally restricts its application in deep-seated tumors, it is ideally suited to image NMSC, where disease morphology and treatment response occur at superficial depths. Leveraging OCTA’s functional insights could thus address the gap in non-invasive yet detailed evaluation of RT’s impact on NMSC lesions.

Parallel advances in deep learning (DL) and artificial intelligence (AI) have propelled medical imaging analysis by capturing subtle, non-linear features often missed by human observers. Although omics-based research has enhanced outcome prediction in various malignancies, these approaches remain underexplored for NMSC imaging. Most DL and omics-focused RT predictors rely on other modalities, such as CT or MRI, and target indications beyond NMSC [14]. Although DL has been used with OCT in retinal disease [15], its potential in NMSC remains largely uncharted, highlighting an opportunity to integrate OCT, AI, and omics data for improved outcome prediction.

The aim of this study is to provide proof-of-concept evidence that an OCTA system, coupled with radiomics and vascular feature analysis, can detect features indicative of vascular structural changes in NMSC patients following radiotherapy.

## 2. Materials and Methods

### 2.1. Patient Cohort

This IRB-approved study (Medical University of Vienna, no. 1246/2013) prospectively enrolled eight patients (March–December 2024) with biopsy-confirmed non-melanoma skin cancer—T1–T2 basal cell carcinoma, T1–T2 squamous cell carcinoma, or actinic keratosis—scheduled for orthovoltage radiotherapy because a multidisciplinary skin-cancer board judged surgery inappropriate owing to expected functional or cosmetic deficit, advanced age, anticoagulation, or serious comorbidities; squamous-cell cases had to be clinically node-negative on physical examination and CT/MRI. Patients were excluded from analysis if the OCTA acquisition was incomplete or of poor quality, if they withdrew early or were non-compliant, or if technical imaging problems compromised data integrity.

### 2.2. Radiotherapy Treatment

All patients were treated with superficial orthovoltage radiotherapy using a Gulmay D3300 orthovoltage therapy unit (Gulmay Medical Ltd., Chertsey, UK). The system features a bi-polar ceramic X-ray tube with an 8 mm focal spot on a tungsten target. Its inherent filtration is 3 mm beryllium, and the accelerating potential can vary from 80 kVp to 250 kVp (0–30 mA), allowing flexibility for customized energy settings based on lesion depth and size.

The clinical target volume included the tumor with a safety margin of 5 mm to 20 mm to encompass and treat subclinical disease. The surrounding tissue was covered with a custom-made lead shield. The dose was prescribed to the surface, and the selection of the dose fractionation schedule was dependent on patients and tumor characteristics such as age, tumor location, size, and thickness. The following fractionation regimens were applied: 50 Gy in 20 fractions, 48 Gy in 16 fractions, 36 Gy in 6 fractions, or 40 Gy in 10 fractions. The radiation schedules are radiobiologically very similar when considering an αβ ratio of 10 Gy.

### 2.3. OCTA Protocol

**Figure 1:**
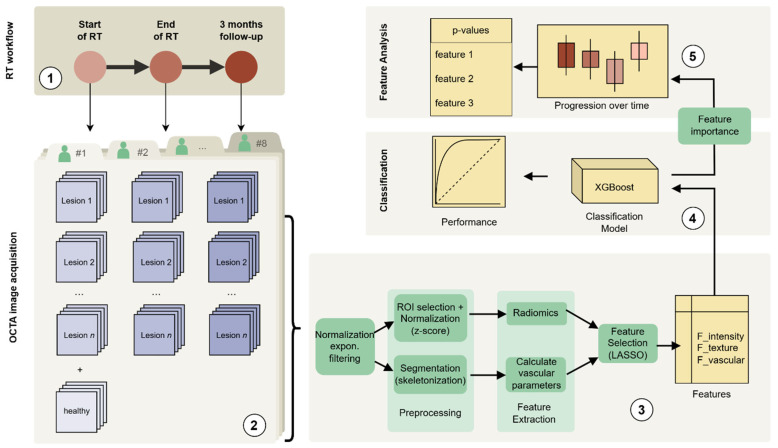
A schematic representation of the data analysis pipeline: (**1**) Radiotherapy regimen and timepoints, (**2**) OCTA image acquisition, (**3**) OCTA image processing and consecutive feature extraction and selection, (**4**) classification modelling based on XGBoost, (**5**) analysis of feature differences across the three timepoints (plus control) including the statistical testing.

A swept-source OCT system was used to non-invasively and label-free capture the morphology of the superficial microvasculature in skin lesions. The light source operates at a central wavelength of 1310 nm and a bandwidth of 29 nm. The imaging system provided a lateral resolution of 31.5 μm, an axial resolution of 27.3 μm, a lateral pixel size of 19.5 μm and an axial pixel size of 13.7 μm. An area of 1 cm^2^ was imaged, providing an imaging depth of 1–1.5 mm where signal-to-decorrelation was robust. The probe was stabilized during use and featured a specialized glass holder that ensures gentle contact with the skin while allowing for easy disinfection and disposal. A glass imaging window provides a flat device skin interface, and a drop of water between the glass and skin improves optical matching [16,17,18]. The OCT system captured structural skin information through backscattered light, and OCTA volumes were generated from four sequential scans at the same site with an intensity-based algorithm [16]. Lesions were scanned using OCTA at three time points: pre-irradiation, immediately post-radiotherapy, and three months after treatment completion. Additionally, a non-malignant spot on the skin close to the lesion for each patient was scanned to approximate healthy local vascular structures (see Figure 1). To minimize patient burden and clinic time, healthy skin was not rescanned at post-RT or 3-month visits.

Each OCTA acquisition encompassed a larger volume, from which a subset of 120 × 512 × 512 pixels (corresponding to 1.15 × 10 × 10 mm^3^, accounting for a skin refractive index of 1.42) was selected. The first dimension represents the imaging depth, corresponding to a value in which vascular contrast is sufficient, and image quality is acceptable for analysis. The light source was calibrated at each use, and a B-scan preview was used as a fast quality check prior to each acquisition to reduce any variations in the OCTA system performance. Artifacts in the raw OCTA volumes were reduced using pixel intensity normalization and exponential filtering to correct motion-related and shadow artifacts. Additional preprocessing steps like 3D median filtering and contrast enhancement were employed to enhance the vasculature, and a depth color coding was used to enable intuitive depth interpretation in 2D OCTA projections. All preprocessing parameters, including the 3 × 3 × 3 3D median filter and contrast enhancement, were fixed across patients and timepoints to ensure reproducibility. Parameter sensitivity was not explored in this pilot dataset but will be systematically evaluated in follow-up work.

### 2.4. Feature Extraction and Selection

Two complementary approaches were used to characterize the OCTA data: (1) the extraction of radiomic features and (2) the derivation of vascular morphology metrics via segmentation and skeletonization (see Figure 1).

For the radiomics part, following data acquisition, the volumetric OCTA images underwent z-score normalization. Central regions of interest (80 × 480 × 480) were then defined within each lesion to minimize artifacts commonly seen near the periphery of OCTA volumes, particularly in the superficial avascular parts. This removed ~16 pixels per side (≈0.3 mm) and 20 pixels from the top and bottom (≈0.4 mm). The volumes were subsequently resampled to half their original size using trilinear interpolation to reduce file size and computational overhead.

From these preprocessed volumes, we computed 18 first-order statistical features (e.g., mean, median, standard deviation, skewness) and 75 texture features: 24 gray-level co-occurrence matrix (GLCM), 14 gray-level dependence matrix (GLDM), 16 gray-level run length matrix (GLRLM), 16 gray-level size zone (GLSZM), and 5 neighboring gray tone difference (NGTDM) features. Shape features were dismissed. This was carried out via the PyRadiomics (v3.1, Python) library. To minimize the directional dependence of texture metrics, all features were extracted in 3D for 13 unique, uniformly distributed directions defined in PyRadiomics, and the resulting values were averaged before statistical analysis. These radiomics features capture global signal distributions and local intensity variations in the OCTA data and are grouped together as *F*_intensity_ and *F*_texture_, respectively.

In the second tier of feature extraction, a methodology developed by Meiburger et al. [13] was used to automatically extract quantitative parameters to describe the vascular network in the OCTA images. The processed OCTA volumes were semi-automatically segmented using Amira (2024, Thermo Fisher Scientific Inc., Waltham, MA, USA) and subsequently skeletonized. Each volume was then compressed into a 2D projection by applying a thresholded weighted average of pixel intensities across all depth levels. Based on the binary masks obtained, quantitative vascular parameters were calculated: number of trees (NT), vascular density (VD), number of branches (NB), max radius, two-dimensional distance metric (DM), inflection count metric (ICM), sum of angles metric (SOAM). In addition, the fractal dimension (FD), entropy, and avascular area (AV) were extracted. These were grouped as *F*_vascular_ for further reference.

In summary, 18 first-order intensity features as *F*_intensity_, 75 higher-order texture features as *F*_texture_ (24 GLCM, 14 GLDM, 16 GLRLM, 16 GLSZM) and 10 vascular features as *F*_vascular_ were extracted, for a total of 103 features.

### 2.5. Classification Modeling

All radiomic features were z-scored and pared down with Least Absolute Shrinkage and Selection Operator (LASSO) with a penalty of λ = 0.001 via 10-fold cross-validation, resulting in a compact subset of features.

To visualize the temporal structure, the selected features were projected onto the first two principal components (PCA). For each cohort—pre-RT, immediate post-RT, 3-month follow-up, and healthy—we plotted the centroid $\left(\bar{x},\bar{y}\right)$ and its 1-standard-deviation ellipse. The ellipse is defined by the eigen-decomposition of the 2 × 2 covariance matrix $\Sigma v_{k}=\lambda_{k}v_{k}$. Its width and height are $2\sqrt{\lambda_{1}}$ and $2\sqrt{\lambda_{2}}$; the tilt angle comes from $v_{1}$. More details on this procedure and the LASSO regression can be found in Appendix C.

Finally, we trained an XGBoost classifier to differentiate the three treatment time points. Performance was estimated with 100 bootstrap out-of-bag resamples, yielding a macro-averaged ROC-AUC (reported as mean ± SD). XGBoost’s gain-based feature importance highlighted the radiomic attributes driving the temporal separation.

### 2.6. Statistics of Feature Analysis for Different Time Points

For the twelve most influential features based on the XGBoost model, overall differences among the three time points plus control categories (healthy, pre, post, and 3M) were evaluated using a non-parametric Kruskal–Wallis test. In this test, all observations were ranked, and the Kruskal–Wallis H statistic was computed to assess whether the distributions of each feature differed significantly among the groups. For Kruskal–Wallis tests, we also report effect size as $\eta_{H}^{2}=(H-k+1)/(n-k)$, where H is the Krukal–Wallis statistc, k the number of groups, and *n* the total observations. Raw *p*-values were adjusted using the Benjamini–Hochberg method, which provides statistical power to detect biologically interesting signals in this exploratory proof-of-concept study while keeping the false-discovery rate explicit and manageable.

For features showing significant overall differences, Dunn’s post hoc test was applied to perform pairwise comparisons between timepoints. This test compared the mean ranks of each pair of groups (with Bonferroni-adjusted *p*-values). Boxplots were then generated to compare metric values across timepoints visually. All statistical analyses were performed using Python 3.8, utilizing the scipy.stats, scikit-posthocs, and statsmodels libraries.

## 3. Results

### 3.1. Cohort Description and OCTA Images

Nine patients with nineteen NMSC lesions were enrolled. One patient was excluded due to incomplete OCTA acquisition at follow-up, leaving eight patients (13 NMSC lesions) for analysis. One lesion was so large that it was scanned multiple times.

**Figure 2:**
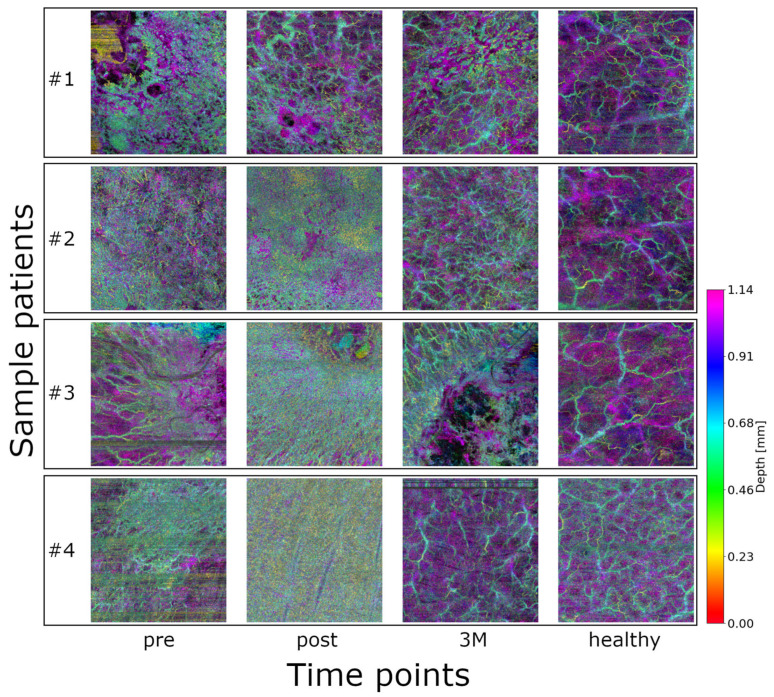
Color-coded depth maps of OCTA volumes from four representative lesions of different patients, shown at the three timepoints (plus control): healthy baseline, pre-treatment, post-treatment, and 3 months post-treatment (3M). An animation of the OCTA scans and the skeletonization can be found in the Supplementary Video S1.

61 image sets (18 volumes at three time points + 7 healthy control) from eight patients with NMSC were initially included in this proof-of-concept study (see Figure 2). Qualitatively, healthy vasculature appears more relaxed and homogeneously distributed. In contrast, the images acquired before radiotherapy show dense and chaotic vascular patterns, which become even more pronounced immediately after treatment. Three months post-radiotherapy, the vasculature still exhibits signs of disorganization but appears noticeably more relaxed compared to both the pre- and immediate post-treatment states. Patient, tumor, and treatment characteristics are summarized in Appendix B.

Although formal clinical–imaging correlations were beyond the scope of this proof-of-concept study, qualitative inspection during routine follow-up generally mirrored the OCTA trends. All patients examined in this study showed regression of the pathology/tumor in response to radiation therapy. No patient showed progression. Lesions showing reduced flow signal and enlarged avascular regions at three months typically appeared more quiescent on visual examination, whereas areas with persistently dense vascular patterns tended to display slower surface recovery. A systematic correlation with clinical endpoints and toxicity data is planned in our ongoing longitudinal extension study.

### 3.2. Feature Selection and Analysis

**Figure 3:**
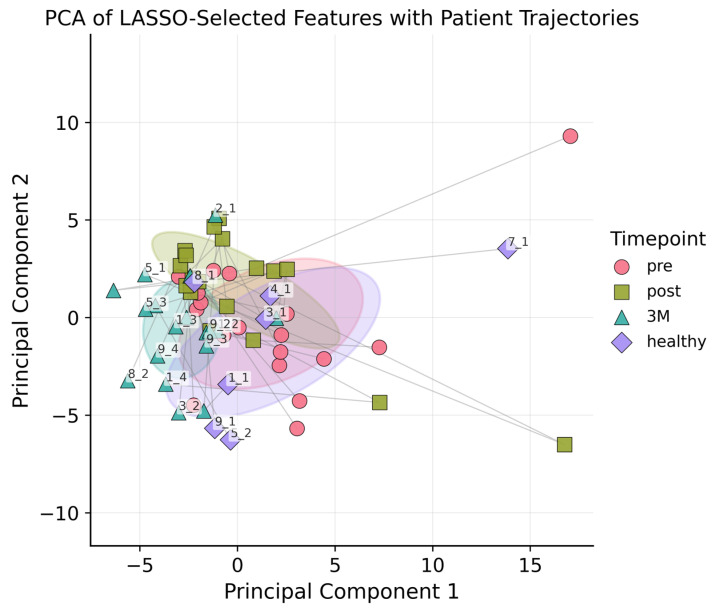
PCA plot with ellipsoids around the centroids of each class (pre, post, 3M, healthy) to indicate 1SD. The first two principal components accounted for 63% of the total variance, and including the third component increased the explained variance to 80%. Each lesion was connected by a line to illustrate its temporal trajectory.

Following LASSO regression, 46 radiomic features were retained: 11 of 18 first-order statistics (e.g., Kurtosis, Median, Robust Mean Absolute Deviation), 13 of 24 GLCM descriptors (e.g., Contrast, Correlation, Inverse Variance, Information Measure of Correlation), 5 of 14 GLDM features (notably Low Gray-Level Emphasis and Dependence Variance), 4 of 16 GLRLM metrics (including Short Run Emphasis and Run Variance), 8 of 16 GLSZM parameters (for example Zone Entropy and Gray-Level Variance), and all 5 NGTDM features (Coarseness, Contrast, Strength, Complexity, Busyness), see also Appendix A. The vascular features were deliberately excluded from the LASSO feature-selection step because they are known to encode mechanistic information about tumor micro-vasculature; consistent with the rationale of Meiburger et al. [13], all vascular metrics were therefore retained in full for subsequent analyses. All features underwent PCA. Figure 3 plots each lesion at baseline, post-RT, and 3 months, revealing tighter clustering at 3 months and hinting at ongoing microvascular or textural changes; nonetheless, PCA alone could not reliably separate the three time points nor the healthy control

### 3.3. Classification Model Performance

**Figure 4:**
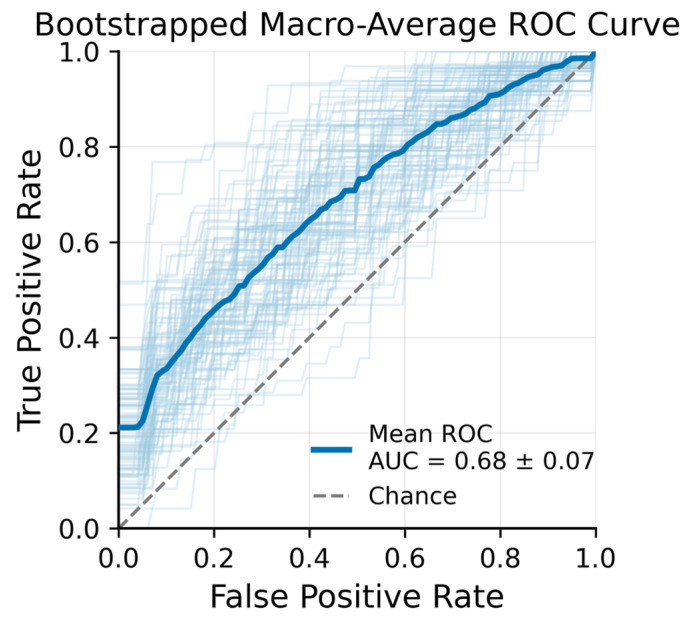
Bootstrapped Macro-Average ROC Curve − XGBoost + LASSO features from 100 bootstrap iterations (light blue lines) with Out-of-Bag (OOB) validation.

An XGBoost classifier was subsequently trained to distinguish OCTA images acquired at the three time points (pre-irradiation, post-radiotherapy, and three months post-treatment). Figure 4 presents the macro-averaged receiver operating characteristic (ROC) curves for this three-class problem. The mean ROC Area Under the Curve (AUC) across bootstrap samples was 0.68 ± 0.07, indicating moderate discriminative performance. XGBoost feature importance analysis identified several top predictors that contributed most strongly to distinguishing the time points.

### 3.4. Longitudinal Feature Analysis

**Figure 5:**
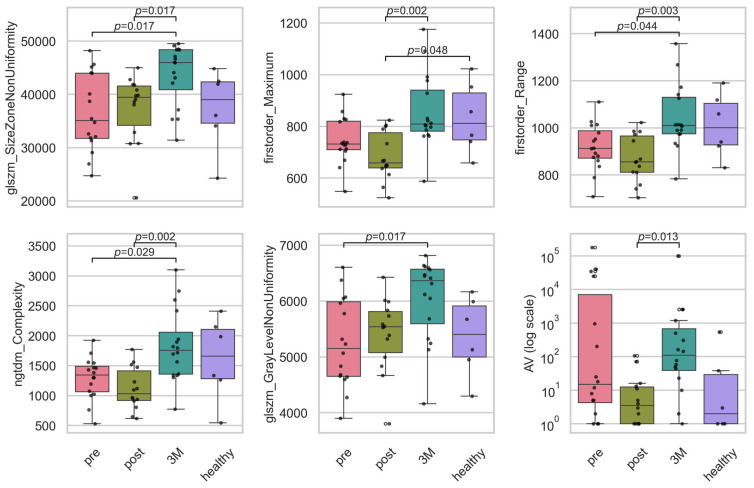
Boxplots of the significant intensity and texture radiomic, and the vascular features. Plotted are the features for the timepoints before RT (‘pre’), directly after RT (‘post’), 3 months after RT (‘3M’) and a healthy skin sample as baseline. Statistically significant pairwise contrasts were annotated with *p*-values (Dunn’s) if the feature was expressing a significant difference (*p* < 0.05) based on the Benjamini–Hochberg adjusted *p*-value of the Kruskal–Wallis test. Boxplots of all 20 important features can be found in Appendix D. The features were size zone non-uniformity (glszm_SizeZoneNonUniformity), maximum intensity (firstorder_Maximum), intensity range (firstorder_Range), complexity (ngtdm_Complexity), gray level non-uniformity (glszm_GrayLevelNonUniformity) and avascular area (AV).

For the longitudinal feature analysis, we retained only features whose XGBoost importance exceeded the average (0.022), thereby reducing our set from 56 to 20 covariates while preserving over 80% of the model’s total importance. Across these features examined (see Figure 5), the Kruskal–Wallis analysis identified six whose distributions changed significantly over the three study time-points plus control after Benjamini–Hochberg correction (q < 0.05) (see Table 1). Three were texture descriptors—glszm_SizeZoneNonUniformity (H = 11.6, $\eta_{H}^{2}$ = 0.178, q = 0.036), ngtdm_Complexity (H = 8.4, $\eta_{H}^{2}$ = 0.113, q = 0.020) and glszm_GrayLevelNonUniformity (H = 8.3, $\eta_{H}^{2}$ = 0.110, q = 0.048)—indicating that 11–18% of the rank variance in these features is attributable to the time-point. Two additional texture features (glcm_MCC and ngtdm_Strength) displayed the largest effects overall ($\eta_{H}^{2}$ ≥ 0.229) but fell just short of the FDR threshold (q = 0.050). Both first-order intensity measures, Maximum (H = 14.1, $\eta_{H}^{2}$ = 0.232, q = 0.020) and Range (H = 10.6, $\eta_{H}^{2}$ = 0.158, q = 0.020), showed robust longitudinal shifts. Within the vascular category, only AV was significant (H = 11.6, $\eta_{H}^{2}$ = 0.179, q = 0.036); all other vascular topology metrics had small effect sizes ($\eta_{H}^{2}$ < 0.06) and non-significant q-values. Together, these results demonstrate that a focused subset of texture and intensity features—and a single vascular measure—undergo moderate-to-large longitudinal changes, whereas the majority of vascular structural parameters remain stable over the course of treatment and follow-up.

## 4. Discussion

This proof-of-concept study is—to our knowledge—the first prospective OCTA base microvascular analysis across three RT timepoints in NMSC, integrating angiographic radiomics with skeleton-based vascular metrics. Our findings suggest that OCTA, combined with advanced feature extraction and machine learning, is capable of identifying microvascular and textural alterations after treatment.

Clinically, this is significant because it points to the potential use of OCTA-based assessments for more nuanced, individualized monitoring of NMSC response to radiotherapy. OCTA could support: (i) baseline phenotyping (e.g., vascular heterogeneity) when surgery is deferred or fractionation choices are being considered; (ii) on-treatment checks in longer regimens (≈10–20 fractions) to flag non-responders early; and (iii) early post-RT surveillance to detect atypical remodeling. In our clinic, scans were obtained alongside routine visits, underscoring workflow compatibility.

OCTA provides repeatable, noninvasive 3D imaging of superficial microvasculature, enabling longitudinal RT monitoring beyond what clinical inspection, dermatoscopy, or routine histopathology can offer. Its main limitations are shallow penetration and motion artifacts, which may degrade image quality and bias quantification. Comparative studies with ultrasound and MRI are needed to define where OCTA adds the most clinical value. The feature trajectories we observed were not only significant but could indicate first-order skin responses to ionizing radiation. Radiotherapy first reduces capillary perfusion [19], which lowers the OCTA signal amplitude—seen as transient drops in Maximum and Range right after treatment. As vessels begin to repopulate, these intensity measures return to their healthy values by three months. In parallel, vessel loss and oedema make flow maps more uneven [20], producing sustained rises in the heterogeneity metrics GLSZM_SizeZoneNonUniformity and GrayLevelNonUniformity. NGTDM_Complexity increases acutely for the same reason but normalizes once the microvasculature reorganizes. Meanwhile, outright capillary dropout enlarges the Avascular Area, which remains elevated throughout follow-up. Thus, each feature potentially tracks physiological sequences: early perfusion loss, persistent structural patchiness, and partial vascular recovery. It is important to note that radiomics measures such as SizeZoneNonUniformity and Complexity describe voxel-level intensity heterogeneity within the OCTA projection, whereas our qualitative assessment of ‘relaxed vasculature’ refers to the overall spatial pattern of perfused vessels. Following radiotherapy, capillary rarefaction (reduced density of microvascular networks) can lead to a patchier signal distribution—hence higher microtexture heterogeneity—even as the macroscopic vascular architecture becomes less tortuous and more regular. This is also consistent with clinical experience, which shows that a response to RT or normalization of normal skin only becomes apparent after at least 3 to 6 months. 

Our results showed that The XGBoost classifier achieved macro-AUC ≈0.68 over 100 bootstrap resamples—hypothesis-generating rather than practice-changing. The feature importance analysis revealed that certain texture and vascular parameters were especially influential in distinguishing time points, suggesting that microvascular remodeling is a key characteristic of radiotherapy response in NMSC.

Although only avascular areas remained significant after FDR correction, OCTA-derived intensity/texture features showed consistent longitudinal shifts which resonate with the qualitative differences that can be seen in Figure 2. These trends justify denser serial OCTA acquisitions and a larger cohort to increase power and formally test whether the observed vascular-feature changes reach statistical significance. Although depth maps appear visually distinct, subjective scoring is vulnerable to observer bias and limited reproducibility. In the future, we propose adding a simple expert-scoring rubric to compare the quantitative findings with human visual assessment. Radiomics, on the other hand, provides standardized, quantitative descriptors that permit effect-size estimation with multiple-testing control and seamless integration into machine-learning models. However, a limitation is the sampling-related uncertainty in radiomics features. While all acquisition parameters were held constant across visits, the hardware and firmware remained unchanged during the study period, and trained operators adhered to a standardized imaging protocol, residual variability cannot be excluded. Future studies will therefore incorporate dedicated test–retest scans and inter-operator variability assessments to formally quantify and minimize these effects.

Other key limitations are the small cohort, exclusion of lesions with poor OCTA quality, OCTA’s shallow penetration and motion sensitivity. Our OCTA protocol targets the superficial microvascular plexus and upper reticular dermis, i.e., ~1–1.5 mm effective angiographic depth, which might not be able to capture the entire depth of remodeled vasculature. In the present study, healthy scans served as baseline context rather than a longitudinal control; future protocols will include repeated healthy-site acquisitions to capture temporal skin changes unrelated to RT and strengthen causal attribution.

However, as a pilot prospective trial, this work essentially supported workflow development and revealed design limitations that directly inform the next study now in planning. Crucially, it showed (A) detectable microvascular change on OCTA and (B) the feasibility of longitudinal tracking across visits. Building on these lessons, the forthcoming study will include clinical endpoints (i.e., local control) and extend follow-up, increase sample size, add site/histology stratification, and incorporate systematic collection of potential confounders with test–retest and inter-operator assessments to enable robust multivariable analyses.

Continued hardware refinements, artifact suppression, and reproducible scan positioning will also be crucial for routine clinical adoption. Residual streaks from motion/phase instability—unavoidable in vivo—may dilute effect sizes, and future protocols will incorporate stricter quality thresholds and test–retest scans to quantify and minimize their impact. With improved lesion masking and complementary structural-OCT radiomics in larger cohorts, we expect greater sensitivity to treatment-related change.

Although the preprocessing pipeline used here was derived from previous segmentation studies [5,13,21], the impact of filter kernels and contrast-enhancement parameters on radiomic feature stability is likely greater, as these features directly depend on voxel-intensity statistics and spatial texture. Future work will include a controlled sensitivity analysis of preprocessing choices on radiomics feature robustness and reproducibility.

Future investigations will additionally incorporate systematic longitudinal profiling of radiation-induced toxicities—captured via our electronic patient-reported outcome (ePRO) platform [22,23]—to explore how early OCTA-derived vascular and texture features relate to clinically meaningful side-effects. Future studies could also benefit from multimodal imaging approaches—such as photoacoustic techniques—to complement OCTA’s structural information with functional readouts of oxygenation and flow within the microcirculatory bed.

## 5. Conclusions

This study demonstrates the potential of OCTA-derived texture and vascular features to detect radiotherapy-induced changes in NMSC lesions at timepoints immediately and 3 months after radiotherapy. While further research is necessary to refine imaging protocols, enhance machine learning frameworks, and validate findings in larger patient cohorts, the non-invasive, label-free nature of OCTA and its ability to capture subtle microvascular changes present a compelling approach to monitoring radiotherapy response in a more personalized and timely manner.

## Figures and Tables

**Table 1:** Results of the intensity/texture radiomic and vascular features. The table shows the Kruskal–Wallis H statistics (KW H-stat) along with the effect size for the Kruskal–Wallis test ($\eta_{H}^{2}$ and the raw (p-value) and Benjamini–Hochberg adjusted significance values (q-value). The Null-hypothesis was rejected for q-values < 0.05.

Feature	Type	KW H-Stat	$\eta_{H}^{2}$	Raw *p*-Value	Benjamini–Hochberg q-Value	$\mathrm{Reject}\;H_{0}$?
original_glcm_Imc1	Texture	9.7	0.139	0.022	0.050	No
**SizeZoneNonUniformity**	**Texture**	**11.6**	**0.178**	**0.009**	**0.036**	**Yes**
DependenceNonUniformity	Texture	8.8	0.121	0.032	0.064	No
Maximal Correlation Coefficient	Texture	14.8	0.246	0.023	0.050	No
Strength	Texture	14.0	0.229	0.018	0.050	No
Zone Variance	Texture	9.6	0.137	0.038	0.068	No
Correlation	Texture	10.1	0.148	0.041	0.068	No
**Complexity**	**Texture**	**8.4**	**0.113**	**0.003**	**0.020**	**Yes**
**Gray Level Non Uniformity**	**Texture**	**8.3**	**0.110**	**0.014**	**0.048**	**Yes**
**Maximum**	**Intensity**	**14.1**	**0.232**	**0.002**	**0.020**	**Yes**
**Range**	**Intensity**	**10.6**	**0.158**	**0.003**	**0.020**	**Yes**
Fractal dimension (FD)	Vascular	3.9	0.019	0.271	0.339	No
**Avascular area (AV)**	**Vascular**	**11.6**	**0.179**	**0.009**	**0.036**	**Yes**
Vascular density (VD)	Vascular	5.8	0.059	0.119	0.184	No
Number of trees (NT)	Vascular	3.1	0.002	0.375	0.417	No
Number of branches (NB)	Vascular	5.6	0.053	0.135	0.193	No
Distance metric (DM)	Vascular	2.4	0.012	0.492	0.518	No
Inflection count metric (ICM)	Vascular	5.2	0.045	0.161	0.214	No
Sum of angles metric (SOAM)	Vascular	3.5	0.010	0.322	0.379	No
Entropy	Vascular	1.7	0.028	0.645	0.645	No

**Figure A1:**
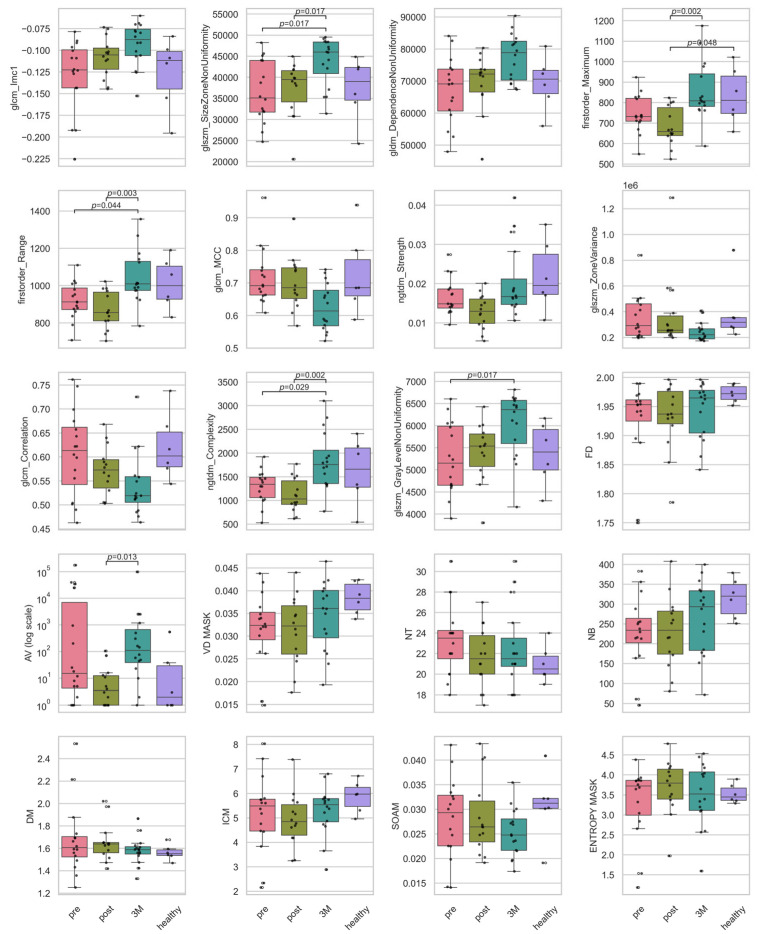
Boxplots of the intensity and texture radiomic, and the vascular features. Plotted are the features for the timepoints before RT (‘pre’), directly after RT (‘post’), 3 months after RT (‘3M’) and a healthy skin sample as baseline. Statistically significant pairwise contrasts were annotated with *p*-values (Dunn’s) if the feature was expressing a significant difference (q < 0.05) based on the Benjamini–Hochberg adjusted q-value of the Kruskal–Wallis test.

## Data Availability

The data presented in this study are available from the corresponding authors upon reasonable request, due to privacy and ethical restrictions.

## Supplementary Materials

The following supporting information can be downloaded at: https://www.mdpi.com/article/10.3390/diagnostics15212698/s1, Video S1: Animation_OCTA_Supplemental.mp4.

## Abbreviations

The following abbreviations are used in this manuscript:

OCTA	Optical Coherence Tomography Angiography
OOB	Out of bag
AUC	Area under the curve
RT	Radiotherapy
NMSC	Non-melanoma skin cancer
LASSO	Least Absolute Shrinkage and Selection Operator
AI	Artificial Intelligence
DL	Deep Learning
CT	Computed Tomography
MRI	Magnetic Resonance Imaging
ePRO	Electronic patient-reported outcome measures

## Appendix A

**Table A1:** Glossary of radiomics and vascular features.

Manuscript Label	Family	Plain-Language Definition
GLCM Informational Measure of Correlation 1 (IMC1) (original_glcm_Imc1)	GLCM (co-occurrence)	An information-theoretic measure of dependency between gray levels derived from the GLCM; captures how much the joint distribution deviates from independence.
Size Zone Non-Uniformity	GLSZM	Variability of zone sizes (contiguous voxels with the same gray level) across the ROI.
Dependence Non-Uniformity	GLDM	Variability of dependence counts (number of neighboring voxels within a given difference) across gray levels.
Maximal Correlation Coefficient (MCC)	GLCM	Nonlinear dependency index based on the second largest eigenvalue of a normalized GLCM matrix.
Strength	NGTDM	Overall prominence of primitives (regions) compared to their neighborhood averages; weighted by occurrence.
Zone Variance	GLSZM	Variance of zone sizes within the ROI.
Correlation	GLCM	Linear correlation of gray levels between neighboring voxels.
Complexity	NGTDM	Weighted complexity of gray-level differences across the image.
Gray Level Non-Uniformity (GLNU)	GLSZM	Variability of gray levels across zones (how unevenly gray levels are represented).
Maximum	First-order	Maximum voxel intensity within the ROI.
Range	First-order	Difference between maximum and minimum intensity within the ROI.
Fractal Dimension (FD)	Vascular (skeleton/graph)	Box-counting estimate of scaling complexity of the vessel network/skeleton (theoretical 1–2 in 2D projections).
Avascular Area (AV)	Vascular (mask)	Fraction (or area) of the ROI without detectable flow signal after binarization.
Vascular Density (VD)	Vascular (mask)	Fraction of ROI occupied by vessel signal (area or volume fraction).
Number of Trees (NT)	Vascular (graph)	Count of connected vascular components in the skeleton graph.
Number of Branches (NB)	Vascular (graph)	Count of branch segments/junction-to-junction edges in the skeleton.
Distance Metric (DM)	Vascular (mask/skeleton)	Mean inter-capillary distance computed from the distance transform of the avascular mask (report in pixels or mm).
Inflection Count Metric (ICM)	Vascular (tortuosity)	Number of inflection points along vessel centerlines normalized by path length.
Sum of Angles Metric (SOAM)	Vascular (tortuosity)	Cumulative absolute turning angle along centerlines normalized by path length.
Entropy	Vascular (distribution)	Shannon entropy of a vessel-derived distribution (e.g., orientation or radius histogram)—specify which in Methods.

## Appendix B

**Table A2:** Patient, tumor and, Treatment characteristics.

Patient, Tumor, and Treatment Characteristics	*n*	%
Gender		
Male	5	56
Female	3	44
Age at diagnosis (years)		
Range	62–100	
Median	82	
Tumor size		
T1	11	85
T2	2	15
Pathology		
BCC	8	62
AK	2	15
SCC	3	23

## Appendix C

In our study, we employed the Least Absolute Shrinkage and Selection Operator (LASSO) for feature selection and regularization among a large set of radiomic features, with the goal of improving the prediction accuracy and interpretability of consecutive models. By imposing an L1 penalty on the regression coefficients, LASSO shrinks some coefficients toward zero.

Before applying the LASSO, radiomic features were standardized to ensure that all predictors contribute equally to the penalty term. The optimal regularization parameter, λ (set to 0.001), which controls the strength of the penalty, was determined via cross-validation:

$$\hat{\beta }={argmin}_{\beta_{0},\beta }\left\{\frac{1}{2n}\sum_{i-1}^{n}{\left(y_{i}-\beta_{0}-\sum_{j=1}^{p}\beta_{j}x_{i,j}\right)}^{2}+\lambda \sum_{j=1}^{p}\left|\beta_{j}\right|\right\}$$

with n as the number of observations and p as the number of features. $x_{i,j}$ is the value of the j-th feature for the i-th observation and $y_{i}$ the corresponding response variable for the i-th observation. As λ increases, more coefficients $\beta_{j}$ are shrunk toward zero, and features with coefficients that become exactly zero are excluded from the final model.

Subsequently, a Principal Component Analysis (PCA) was performed to visualize and understand structural differences among the three time points (pre-irradiation, immediately post-radiotherapy, and three months post-radiotherapy).

For each group (pre, post, 3 months and healthy), the centroid $\left(\bar{x},\bar{y}\right)$ was determined with an ellipsoid indicating the first standard deviation of that distribution.

$$\bar{x}=\frac{1}{N}\sum_{i=1}^{N}x_{i}\;,\;\bar{y}=\frac{1}{N}\sum_{i=1}^{N}y_{i}$$

with $x_{i}$ and $y_{i}$ corresponding to the first (PC1) and second (PC2) principal component values, respectively, for all N PCA-transformed points. To characterize the spread of the data, we perform an eigen decomposition of the covariance matrix Σ. The eigenvalue problem is given by:

Σv= λv

where $\lambda_{1}$ and $\lambda_{2}$ are the eigenvalues and $v_{1}$ and $v_{2}$ are the corresponding eigenvectors. These eigenvalues represent the variances along the principal axes. For plotting the ellipse corresponding to one standard deviation σ, the width w and height h of the ellipse and the orientation angle θ, determined by the eigenvector are set as:

$$w=2\sigma_{1}=2\sqrt{\lambda_{1}},\;h=2\sigma_{2}=2\sqrt{\lambda_{2}},\;\theta =\tan^{-1}\left[\begin{array}{c} v_{1,1}\\ v_{1,2} \end{array}\right]$$

To explore whether there were some underlying changes on micro-vascular level, expressed in the extracted features, an XGBoost model [24] was then trained to classify OCTA images based on their time point. Model performance was assessed via bootstrap resampling (*n* = 100) with Out-of-Bag (OOB) validation. Receiver Operating Characteristic (ROC) curves were generated from these OOB samples, and a macro-averaged Area Under the Curve (AUC) was reported (mean ± standard deviation). XGBoost feature importance was also computed to identify the most influential features differentiating the time points.
